# Enamel Remineralization Potential of Conventional and Biomimetic Toothpaste Formulations: A Comparative In Vitro Study

**DOI:** 10.3390/dj14020082

**Published:** 2026-02-02

**Authors:** Cristina-Angela Ghiorghe, Ionuţ Tărăboanţă, Sorin Andrian, Galina Pancu, Corneliu Munteanu, Bogdan Istrate, Fabian Cezar Lupu, Claudia Maxim, Ana Simona Barna

**Affiliations:** 1 Grigore T. Popa University of Medicine and Pharmacy, 16 Universitatii Str., 700115 Iasi, Romania; 2 Mechanical Engineering, Mechatronics and Robotics Department, “Gheorghe Asachi” Technical University of Iasi, 63 D Mangeron Blvd, 700050 Iasi, Romania; 3 Technical Science Academy Romania, 26 Dacia Blvd, 030167 Bucharest, Romania; 4 Faculty of Chemical Engineering and Environmental Protection “Cristofor Simionescu”, “Gheorghe Asachi” Technical University of Iasi, 71 A Mangeron Blvd, 700050 Iasi, Romania

**Keywords:** enamel remineralization, fluoride, NovaMin, nano-hydroxyapatite, arginine, SEM–EDX, Vickers microhardness

## Abstract

**Background/Objectives**: Dental caries remains one of the most prevalent chronic diseases worldwide, making enamel remineralization a key objective in minimally invasive dentistry. This in vitro study compared the remineralization efficacy of five therapeutic toothpastes containing fluoride, NovaMin, CPP-ACP, nano-hydroxyapatite, arginine, and xylitol. **Methods**: Sixty enamel specimens were prepared from extracted human posterior teeth and artificially demineralized. Samples were randomly allocated into six groups (n = 10): one negative control (C1) stored in artificial saliva and five treatment groups (P1–P5). A 28-day remineralization protocol with twice-daily applications was performed. Scanning electron microscopy (SEM) and energy-dispersive X-ray spectroscopy (EDX) were used to assess surface morphology and elemental composition (Ca, P, F, Na, O, Ca/P ratio) at days 1, 14, and 28. Vickers microhardness testing was used to evaluate changes in mechanical properties. Statistical analysis included one-way ANOVA, repeated measures ANOVA, Tukey’s post hoc test, and Kruskal–Wallis where appropriate (α = 0.05). **Results**: All therapeutic toothpastes produced some increase in mineral content compared to the demineralized control. At day 28, significant intergroup differences were observed for calcium, phosphorus, and fluoride (*p* < 0.001). The arginine–fluoride formulation (P4) and the NovaMin-based formulation (P3) showed the most consistent increases in Ca and P, with SEM revealing the formation of a continuous, compact surface layer and marked reduction in prismatic porosities. Fluoride-containing toothpastes (P1, P3, P4) showed significant fluoride incorporation (*p* < 0.001 vs. control). The nano-hydroxyapatite/xylitol prototype (P5) produced a delayed but progressive increase in Ca and P, with partial filling of prismatic spaces. The CPP-ACP-based toothpaste (P2) led to limited changes, with only slight differences vs. control at day 28. Vickers microhardness values increased significantly in groups P1, P3, P4, and P5 (*p* < 0.05), in agreement with the higher mineral levels found in these samples. **Conclusions**: Under the present in vitro conditions, toothpastes containing fluoride in combination with NovaMin or arginine, as well as nano-hydroxyapatite/xylitol, demonstrated the highest remineralization potential under the present in vitro conditions, both chemically and mechanically. Xylitol-based formulations without a direct mineral supply showed limited effects. The pH and active composition of the toothpaste strongly influenced enamel remineralization outcomes.

## 1. Introduction

The remineralization potential of dental enamel is a central topic in dental caries prevention, given the still high prevalence of this condition worldwide. Dental caries remains one of the most widespread chronic diseases worldwide, affecting over 3.5 billion people and considered the most common non-communicable disease [1]. From an etiological point of view, dental caries is a multifactorial condition resulting from the complex interaction between acidogenic bacteria in dental plaque, frequent consumption of fermentable carbohydrates, and environmental and host factors. The organic acids produced by bacteria cause progressive demineralization of the hard structure of the tooth, marking the onset of the carious process [2]. Early enamel lesions, known as white spots, can be reversible if the balance between demineralization and remineralization processes is restored, which is essential in modern minimally invasive therapeutic approaches [3].

Contemporary strategies for preventing dental caries focus on promoting remineralization and reducing demineralization through the use of topical agents included in toothpastes. Fluoride remains the gold standard in caries prevention, with the ability to reduce enamel solubility through the formation of fluorapatite and to inhibit the metabolism of cariogenic bacteria [4]. In parallel, compounds such as biomimetic hydroxyapatite, amorphous calcium phosphate, bio-glass, and casein derivatives have been introduced as alternative or complementary remineralizing agents to fluoride [5]. Xylitol, a non-fermentable sweetener, is recognized for its ability to inhibit the growth of Streptococcus mutans, and enzymes such as lactoperoxidase, glucose oxidase, and lysozyme contribute to the balance of the oral microbiota [6]. Modern toothpaste formulations thus seek to synergize these components to increase the effectiveness of remineralization in the context of daily oral hygiene. In addition, xylitol is able to form soluble complexes with calcium ions, thereby stabilizing them and facilitating their diffusion into demineralized enamel areas, which supports remineralization processes even in the absence of bacterial biofilm [7].

From a clinical point of view, the development of effective remineralization products is essential for reducing the incidence of caries and promoting minimally invasive dentistry. Despite the widespread use of fluoride, the prevalence of caries remains high, especially in children and adolescents, suggesting the need for additional interventions [7,8]. Preventing the progression of early lesions is less costly and less invasive than treating advanced caries. In this regard, therapeutic toothpastes can become an important link in the non-invasive management strategy of dental caries, allowing early intervention on the mineral balance of the enamel [8].

The toothpaste formulas tested in this study contain various combinations of active agents, each with a specific role in enamel remineralization. Fluoride contributes to the formation of a mineral structure that is more resistant to acids, while biomimetic hydroxyapatite promotes mineral deposition on the surface of sub-surface lesions, contributing to the restoration of the enamel microstructure [5,9]. Xylitol reduces acidogenic bacterial activity and stimulates remineralization by increasing the local pH [6]. Enzymes added to some toothpastes support the natural immune defense of the oral cavity, reducing microbial colonization and indirectly supporting the remineralization process [10]. *Lithothamnion calcareum*, a red marine algae rich in calcium carbonate, magnesium, and trace elements, has been reported to support enamel mineralization through biomimetic ion supply [11]. Thus, these products can offer a combined therapeutic effect with significant benefits in caries prevention.

The practical motivation for the study stems from the need to evaluate the real effectiveness of these formulas under controlled conditions in order to ground evidence-based clinical recommendations. Preventive dental practice requires products that not only clean the tooth surface but also actively contribute to its restoration. Especially for patients with an increased caries risk, daily use of a remineralizing toothpaste could prevent the onset or worsening of carious lesions [10].

Despite the growing number of studies on enamel remineralization, a clear methodological gap remains. Specifically, direct comparative in vitro evaluations of conventional fluoride and biomimetic toothpaste formulations under identical experimental conditions, combining SEM–EDX and mechanical assessment, are still scarce. In addition, toothpaste formulations containing enzyme-based systems or marine-derived mineral sources such as *Lithothamnion calcareum* are increasingly used in clinical practice; however, their inclusion in comparative in vitro remineralization studies remains limited, and their physicochemical effects on demineralized enamel surfaces, independent of antibacterial activity, are not clearly established. In the present in vitro model, antibacterial effects were not investigated; enzyme- and arginine-containing formulations were included to assess their indirect physicochemical influence on enamel remineralization. To address this gap, the aim of the present study was to evaluate the remineralizing potential of therapeutic toothpastes containing fluoride, NovaMin, nano-hydroxyapatite, xylitol, CPP-ACP, and arginine on in vitro simulated incipient enamel lesions. The null hypotheses were that: (i) no significant differences would be found among the tested toothpastes regarding their effect on enamel remineralization, and (ii) none of the formulations would produce significantly different remineralization compared to the negative control group maintained in artificial saliva.

## 2. Materials and Methods

### 2.1. Sample Selection and Preparation

The study was carried out in compliance with the ethical standards outlined in the Declaration of Helsinki and received approval from the Ethics Committee of the Grigore T. Popa University of Medicine and Pharmacy, Iași, Romania (Approval No. 633/Approval date: 31 July 2025).

Prior to the experimental procedures, the sample size calculation was performed using G*Power software, version 3.1.9.7 (Heinrich Heine University Düsseldorf, Düsseldorf, Germany). Based on a moderate effect size (Cohen’s d = 0.5), a power of 0.80, and a significance level of 0.05, the analysis indicated that a minimum of 60 samples were required. Vickers microhardness (VHN) was considered the primary outcome of the study, as it reflects the functional mechanical recovery of enamel. A moderate effect size (Cohen’s d = 0.5) was selected based on previously published in vitro enamel remineralization studies reporting comparable differences in microhardness values.

**Figure 1:**
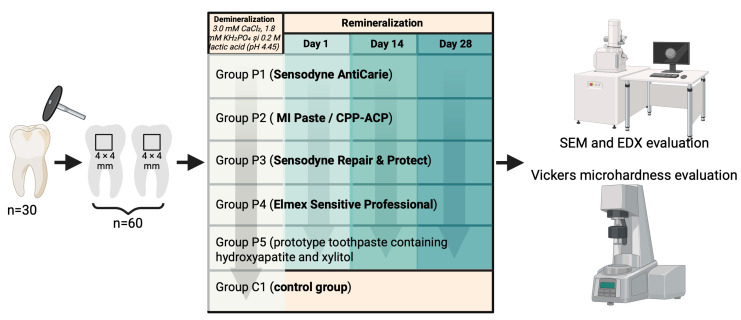
Study design.

Thirty extracted human posterior teeth were initially selected for this in vitro study. The teeth were obtained from patients who underwent extractions for orthodontic or periodontal reasons, after obtaining informed consent and in full compliance with ethical and biosafety guidelines. The inclusion criteria required that all teeth have intact coronal structures, free of caries, fractures, enamel cracks, structural anomalies, signs of demineralization, or previous restorative interventions, to ensure sample uniformity and reliable results. The working protocol is illustrated in Figure 1.

After extraction, the teeth were cleaned with distilled water in an ultrasonic bath (Evo Sonic 2.5 L, EvoDent, Arad, Romania) and stored in distilled water at room temperature (≈23°C) to prevent dehydration until processing.

Each tooth was sectioned in the vestibulo-oral direction using a microtome for hard tissues (Microcut 152, Metkon Instruments Inc., Bursa, Turkey), obtaining two flat proximal enamel surfaces (mesial and distal) located near the enamel–cement junction. This process yielded a total of 60 individual enamel samples.

The prepared enamel surfaces were polished under constant water irrigation using silicon carbide abrasive discs with increasingly finer grit sizes (P320, P600, and P1200) to obtain a standardized, smooth surface suitable for treatment.

An acid-resistant lacquer was applied to each specimen to delimit a controlled treatment area, leaving a 4 mm × 4 mm window of exposed enamel that served as the region of interest for all experimental procedures. The 60 enamel samples were then randomly allocated into six groups of 10 specimens each (n = 10). Randomization was achieved by assigning each sample a unique identification code and distributing them into groups using a random number table, ensuring equal allocation probability. Group C1 (control) underwent only demineralization stage and was subsequently stored in artificial saliva for the duration of the study, without exposure to remineralization protocols. Moreover, the study groups P1–P5 were subjected to demineralization procedure, followed by remineralization treatments using different toothpaste formulations. The specific products and their compositions are detailed in Table 1.

### 2.2. Artificial Caries Simulation

All samples were subjected to artificial demineralization by immersion for 72 h in an acid-based solution composed of 3.0 mM CaCl_2_, 1.8 mM KH_2_PO_4_, and 0.2 M lactic acid (pH 4.45) [12] at a constant temperature of 37 °C in an incubator (Biobase BJPXH30II, Biodusty, Shandong, China). The samples were then rinsed with distilled water and stored in artificial saliva prior to remineralization. The pH of the demineralization solution and each toothpaste sample was measured using a calibrated portable pH meter (Thermo Scientific Eutech pH 5+, Thermo Fisher Scientific, Vernon Hills, IL, USA).

The pH values of each tested toothpaste solution are presented in Table 2. Toothpaste slurries were freshly prepared by mixing one part toothpaste with three parts distilled water (1:3, *w*/*v*).

### 2.3. Remineralization Procedure

Remineralization cycles were performed twice a day for 28 consecutive days. Each cycle consisted of rinsing the sample with distilled water, followed by application of the corresponding tested toothpaste using a microbrush for 2 min. At the end of the remineralization stage, the samples were stored in artificial saliva at a constant temperature of 37 °C.

No bacterial biofilm was included in the experimental design; therefore, the model evaluated exclusively chemical demineralization–remineralization processes.

### 2.4. SEM and EDX Analysis

Surface morphology was examined using a scanning electron microscope (Quattro C, ThermoFisher Scientific, Waltham, MA, USA) operated in high-vacuum mode at 15 kV accelerating voltage, 10 mm working distance, and a secondary electron (SE) detector. Images were captured at magnifications ranging from 500× to 5000×. All specimens were air-dried and mounted on aluminum stubs using carbon adhesive tabs; no additional surface coating was applied. SEM images were obtained at three time points: after the first day of remineralization treatment and after day 14 and day 28 of treatment.

Elemental analysis was performed using energy-dispersive X-ray spectroscopy (EDX), also on the Quattro C scanning electron microscope (SEM), Thermo Scientific Quattro C, Brno, Czech Republic, equipped with the EDAX (Energy-Dispersive X-Ray Spectroscopy, Thermo Scientific Quattro C, Brno, Czech Republic) system. The system operated automatically with the selection of the list of elements, applying point, linear, and mapping scans to identify and locate calcium, phosphorus, oxygen, fluoride, and sodium.

The measurements were repeated three times on different areas to obtain the mean values. Detection was performed in Automatic-Precise mode with PB-ZAF correction and a sensitivity of 0.01%. Standard deviations were calculated from three determinations in an area of 0.25 mm^2^ for each element. The absolute and relative errors of the detector were also reported for comparison of the results.

SEM–EDX analyses at days 1, 14, and 28 were performed on the same enamel specimens, using different, non-overlapping areas within the predefined treatment window, in order to avoid beam-induced artifacts or surface alteration.

### 2.5. Vickers Microhardness Evaluation

Surface microhardness was evaluated using a microindentation protocol performed on a tribometer (CETR UMT-2, Bruker Corporation, Berlin, Germany) equipped with a Vickers diamond indenter (pyramidal geometry with an apex angle of 136°).

For each enamel specimen, five indentations were placed at least 100 μm apart and at least 200 μm from the margins to avoid edge effects. Indentation was performed under a 1 N load with a dwell time of 15 s, following a standardized loading–holding–unloading sequence as provided by the instrument.

The diagonals of each indentation were automatically measured using the integrated optical/imaging system of the tribometer software, and hardness was expressed as Vickers Hardness Number (VHN) according to the classical Vickers formula.

For each specimen, the mean of the five measurements was recorded as the final VHN value.

### 2.6. Statistical Analysis

The statistical analyses of the obtained VHN values and EDX data (At.%) of each analyzed element were performed using IBM SPSS Statistics, version 29.0.0 (IBM Corp., Armonk, NY, USA), with the significance threshold set at *p* = 0.05. The normality of the data distribution was evaluated using Shapiro–Wilk test, while the homogeneity of the variances was assessed using Levene test. Based on the results of these preliminary tests, parametric (one-way ANOVA followed by Tukey’s post hoc test) or nonparametric (Kruskal–Wallis) methods were applied to evaluate intergroup differences between the experimental groups (P1–P5) vs. the control group (C1) on days 1, 14, and 28. To evaluate intragroup changes over time, Repeated Measures ANOVA test, followed by post hoc Tukey, was used. These analyses allowed the identification of statistically significant variations in elemental concentrations both between the tested formulations and within them during the experimental period.

## 3. Results

**Figure 2:**
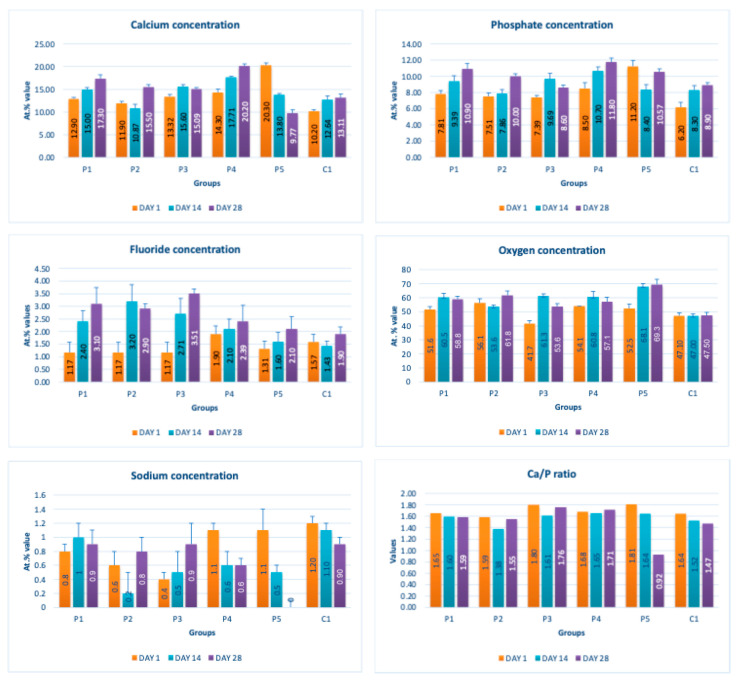
Mean values ± standard deviations for each of the evaluated elements (calcium, phosphorus, fluoride, oxygen, sodium, Ca/P ratio) within each group (C1, P1–P5) and each evaluation time (Day 1, Day 14, and Day 28).

Figure 2 presents the mean values and standard deviations for each of the evaluated elements (calcium, phosphorus, fluoride, oxygen, sodium, Ca/P ratio) within each group (C1, P1–P5) and each evaluation time (Day 1, Day 14, and Day 28).

Throughout the evaluation period, all experimental groups generally recorded higher calcium concentrations compared to the negative control group, C1 (11.98 ± 0.68 At.%). The highest value was recorded in group P4 (Elmex Sensitive Professional), with a maximum of 20.20 ± 0.35 At.% on day 28, indicating superior and consistent remineralization. Group P1 (Sensodyne AntiCarie) reached the highest value (17.30 ± 0.89 At.%) on day 28, and P3 (Sensodyne Repair & Protect) recorded the highest value of 15.60 ± 0.45 on day 14. P2 (CPP-ACP) recorded 11.90 ± 0.46 At.% on day 1, followed by a decrease on day 14 and an increase to 15.50 ± 0.81 on day 28. P5 had an initial peak of 20.30 ± 0.50 but decreased steadily to 9.77 ± 0.76, suggesting a transient effect and high variability. Statistical analysis revealed significant differences between the control group and most treatments on day 28 (*p* < 0.001 for P4; *p* = 0.003 for P1; not significant for P3). Evolutionarily, all groups except the control showed significant increases between days 1 and 28 (*p* < 0.001 for P1, P3, P4, and P5; *p* = 0.027 for P2).

For phosphorus, groups P4 and P5 consistently exceeded the control, reaching values of 11.80 ± 0.46 At.% on day 28 (P4) and 10.57 ± 0.33 At.% (P5), with P1 recording values of 10.90 ± 0.70 At.%. P4 stood out for its high levels and low variability. Significant differences were observed between the control and P4 (*p* < 0.001), P1 (*p* = 0.003) and P5 (*p* = 0.011). All treated groups, except for the control, showed significant increases between days 1 and 28 (*p* < 0.001), and P5 showed a delayed but sustained effect (*p* = 0.025 between days 14 and 28).

In the case of fluoride, the control group showed the lowest mean values on each testing day. P3 reached the highest levels (3.51 ± 0.17 At.% on day 28), followed by P1 (3.10 ± 0.65) and P2 (2.90 ± 0.19), all with significant increases compared to the control (*p* < 0.001) and an upward trend (*p* < 0.001). P4 showed a moderate increase (mean: 2.13 ± 0.46) without statistical significance (*p* > 0.05), and P5 increased modestly but significantly between days 1 and 28 (*p* = 0.005; final: 2.10 ± 0.49).

Oxygen values ranged from 41.7 ± 2.1 to 56.1 ± 3.1 on day 1, from 54.6 ± 2.4 to 68.1 ± 3.3 on day 14, and from 53.6 ± 2.9 to 69.3 ± 3.1 on day 28. Significant differences were observed in P3 and P5 between day 1 and days 14/28 (*p* ≤ 0.007), as well as between the control and P1, P3, P4, and P5 on day 14 (*p* = 0.001) and between the control and P1, P2, and P5 on day 28 (*p* = 0.001).

Sodium At.% values varied between 0 and 1.20, with significant differences on day 28 between control and P4 (*p* = 0.028) and P5 (*p* = 0.001), respectively.

The Ca/P ratio ranged between 0.92 and 1.81, with significant differences in P2 between days 1 and 14 (*p* = 0.006) and in P5 between days 1/14 and 28 (*p* = 0.001).

The SEM images in Table 3 showed distinct morphological changes in the dental enamel depending on the treatment applied and the evaluation interval, outlining a differentiated evolution of the remineralization process.

SEM examination revealed clear morphological differences among groups and across timepoints. In the control group, demineralized enamel maintained a honeycomb-like appearance throughout the experiment, with visible prismatic dissolution. In group P1 (fluoride), a discontinuous superficial layer with amorphous deposits was observed at day 1, becoming more uniform by day 28, with partial reduction in surface porosities. Samples treated with MI Paste (CPP–ACP) exhibited early deposition of fine granular mineral clusters within interprismatic regions, producing partial pore filling and subtle surface smoothing by day 14. By day 28, a more uniform superficial layer was observed, although prismatic outlines remained distinguishable, consistent with the gradual infiltration of amorphous calcium phosphate complexes. Samples in group P3 (NovaMin) showed abundant surface deposits from day 1 and a continuous compact layer with reduced porosity by day 28. Group P4 (arginine + fluoride) also showed early mineral deposits that progressively increased in density, with a more homogeneous surface after 28 days. Group P5 (nano-hydroxyapatite/xylitol) exhibited partial pore filling at day 1 and gradual surface smoothing, although residual prismatic outlines remained visible at day 28.

Microhardness results at days 1, 14, and 28 are presented in Table 4. The demineralized control group (C1) showed consistently low hardness values between 212 ± 18 and 219 ± 14 VHN, with no significant changes over time (*p* > 0.05). All treatment groups demonstrated progressive increases in hardness, with marked intergroup differences (ANOVA, *p* < 0.001).

The NovaMin-containing toothpaste (P3) produced the highest recovery, reaching 405 ± 38 VHN at day 28. Notable improvements were also observed in the arginine–fluoride toothpaste (group P4) with significant increases at day 14 and 28 vs. day 1 (*p* < 0.01). The conventional fluoride toothpaste (group P1) showed significant differences after 14 and 28 days of application vs. day 1 (*p* < 0.01) and at day 28 vs. C1 (*p* < 0.001). The hydroxyapatite–xylitol paste (P5) recorded values between 117 ± 9 on day 1 and 335 ± 16 VHN on day 28 and showed significant differences when compared to control group C1 on day 28.

Intragroup comparisons revealed significant increases between days 1 and 28 in groups P1, P3, P4, and P5 (*p* < 0.01). Post hoc analysis confirmed that P3 exceeded all other groups at day 28 (*p* < 0.01).

## 4. Discussion

In this in vitro study, we evaluated the remineralization potential of dental enamel by five toothpaste formulations based on fluoride, nanohydroxyapatite, xylitol, Recaldent, and NovaMin technology. All formulations demonstrated some degree of mineral restoration of incipient enamel lesions, but the efficacy varied depending on the composition of the active ingredients and the physicochemical properties (such as pH) of the toothpastes. The elemental values (At.%) obtained by EDX (Ca, P, F, Na, O and Ca/P ratio) reflected significant differences between groups, suggesting distinct mechanisms of action. Comparison of SEM images of the treated surfaces also revealed notable morphological differences between groups, correlated with the degree of remineralization achieved.

Fluoride remains the gold standard in the prevention and remineralization of early caries lesions, with well-known mechanisms. Its ions bind to the crystals in the demineralized enamel subsurface, forming fluorapatite, which is more resistant to acids than hydroxyapatite [13,14]. At the same time, it can inhibit the metabolism of biofilm bacteria by forming intracellular hydrofluoric acid, which reduces the production of cariogenic acids [15]. In our study, fluoridated toothpastes, regardless of the type of fluoride, significantly increased the fluoride ion content in enamel, creating a reservoir of protective compounds. Even at low levels, fluoride in biofilm and saliva has a cumulative remineralizing effect, thus explaining the clinically reported reduction in caries incidence [16,17]. Fluoridated toothpastes had varying pH values (between 6.24 and 8.81), so the effect was due to the chemical action and available ions and not to pH changes. Thus, the obtained results confirm the effectiveness of fluoride in restoring enamel and forming protective compounds.

Nanohydroxyapatite is increasingly proposed as a biological alternative to fluoride due to its ability to integrate directly into the enamel structure and fill the microscopic pores of incipient lesions [18]. Research has shown that hydroxyapatite nanoparticles form a protective layer and provide the minerals necessary for the restoration of demineralized areas [19]. An in vitro study on temporary teeth even observed a higher mineral intake in subjects treated with nanohydroxyapatite alone compared to those treated with fluoride or fluoride + nanohydroxyapatite, although the differences were not statistically significant [20]. In our study, the nanohydroxyapatite-based paste prototype caused an increase in Ca and P in the enamel after demineralization, confirming its remineralizing contribution. SEM analysis of the enamel surface showed clear changes, in which the enamel prisms, initially with demineralized areas, showed internal mineral deposits, better-defined contours, and partially filled voids [20,21]. This is explained by the fixation of hydroxyapatite crystals on the lesion, which act as crystallization nuclei for Ca and P in saliva. Although the effect is slightly inferior to fluoride in the short term, the results are promising, especially since recent studies show comparable efficacy to fluoride-based pastes in preventing caries [19,22,23].

The incorporation of *Lithothamnion calcareum*, a natural marine mineral source rich in calcium carbonate, magnesium, and bioactive trace elements, into toothpaste formulations enhances the remineralizing effect of biomimetic nano-hydroxyapatite by providing complementary essential ions involved in apatite crystal nucleation and growth. Studies demonstrate that this synergy promotes the regeneration of enamel and dentin structures, effectively replicating the properties of hard dental tissues. Due to its high mineral content, *Lithothamnion calcareum* shows enamel remineralization effects comparable to fluoride but without the risks linked to fluoride overexposure, positioning it as a natural and effective alternative in oral care products [11].

As for NovaMin technology, a calcium and sodium phosphosilicate, it releases calcium and phosphate ions upon contact with saliva, forming precipitates that seal and remineralize the enamel. In the group treated with NovaMin-based toothpaste, EDX elemental analysis showed the highest increases in Ca and P of all formulations, indicating massive mineral deposition. Another in vitro study confirms these results, reporting a significant increase in the Ca/P ratio after only 10 days of treatment [24]. Sodium ions in bio-glass increase the local pH, creating an alkaline environment favorable to the formation of carbonated hydroxyapatite [25]. In our study, the slightly alkaline pH of the paste (pH value of 8.81) further supported the process. The images obtained by SEM analysis showed a smooth, homogeneous surface without porosity, a sign of almost complete remineralization, similar to the results obtained in another similar study [26]. Recent studies confirm the superiority of NovaMin over conventional fluoride pastes in increasing the mineral content and microhardness of enamel [24,25]. Thus, NovaMin acts effectively both by directly supplying ions and by optimizing the pH for enamel restoration.

MI Paste (CPP–ACP) demonstrated a moderate remineralization effect, consistent with the ability of casein phosphopeptides to stabilize and deliver amorphous calcium phosphate nanoclusters into enamel microporosities. The progressive increases in Ca, P, and Ca/P ratios align with previously reported improvements in surface microhardness and subsurface lesion repair. CPP–ACP has been shown in clinical and in vitro studies to enhance mineral deposition and reduce lesion depth, particularly in early enamel caries. Our findings support this mechanism, showing partial pore occlusion and a gradual smoothing of the enamel surface [26,27].

Xylitol, a sugar alcohol with an anticariogenic effect, does not have the ability to directly add minerals but creates conditions that support natural remineralization, as it is not fermented by cariogenic bacteria and thus reduces acid production [6]. In addition, it maintains the pH at a neutral value and can stimulate salivary flow, increasing the availability of Ca and P ions. In our study, under in vitro conditions, without the presence of cariogenic bacteria but with pH variations, toothpaste with xylitol produced a modest increase in Ca and P, probably due to the absence of bacterial biofilm. In contrast, a previous study presented a toothpaste formula with xylitol and natural plant extracts that generated more effective remineralization than even a fluoride-based toothpaste [26], with significant increases in Ca and P [6,27,28]. SEM analysis revealed smooth and intact enamel surfaces, probably due to the high pH that favors the precipitation of minerals from artificial saliva. Clinically, xylitol is best known for its preventive properties, reducing the acidogenicity of biofilm and the incidence of caries. The results of our study show that, although it does not remineralize directly like fluoride or HA, xylitol supports natural processes and may be a valuable adjuvant, especially in children and patients at increased risk of caries.

Arginine, present in the Elmex Sensitive Professional formula, is characterized by a dual beneficial action in the prevention and treatment of incipient carious lesions. On the one hand, the metabolism of arginine by arginolytic oral bacteria produces ammonia, which increases the local pH and neutralizes the acids in the biofilm, thus reducing the solubility of the enamel [29,30,31]. On the other hand, the combination of arginine with sources of calcium and fluoride promotes the precipitation of mineral ions on demineralized surfaces, accelerating the process of restoring the prismatic-crystalline structure [32]. The results obtained in this study support these mechanisms, with the group treated with arginine and fluoride-based paste achieving consistent increases in calcium and phosphate content, along with a visible reduction in porosity on SEM analysis, confirming the effectiveness of this combination in enamel remineralization and oral environment stabilization.

Therefore, elemental analysis confirmed the enrichment of enamel with minerals after 28 days of treatment with various types of toothpaste, supporting the qualitative observations. Formulas with calcium and phosphate sources, such as nanohydroxyapatite, generated significant increases in Ca and P compared to demineralized enamel [27,33]. Hydroxyapatite also provided mineral input, but more moderately, possibly through the formation of a surface layer [22]. Xylitol toothpaste, although without mineral additives, still produced a slight gain in Ca and P, probably by maintaining a neutral pH and through any trace elements in its composition [16]. Fluoride was detected only in the groups containing it, sufficient to incorporate measurable amounts of F into the enamel [34]. It can appear as fluorapatite in the crystal structure or as superficial calcium fluoride, both of which provide acid protection. Overall, bio-glass and hydroxyapatite restored the enamel composition as close as possible to the physiological one, and fluoride added additional protection. The initially high calcium values in the P5 group likely reflect early surface deposition of nano-hydroxyapatite rather than stable mineral incorporation. Partial removal of loosely bound deposits during immersion in artificial saliva may account for the lower Ca levels detected by EDX at later time points.

The pH of toothpastes plays a key role in the demineralization–remineralization balance, with a neutral or alkaline value favoring the deposition of Ca and P ions from saliva. The tested pastes had varying pH values, depending on their composition. Alkaline formulas, such as those with amorphous calcium phosphate or bio-glass [17], create an optimal environment for remineralization. In our study, the prototype toothpaste had an alkaline pH due to calcium carbonate, which acts as a buffer and releases Ca^2+^ in an acidic environment [35]. Along with this, NovaMin technology (with Na_2_O from bio-glass) had the best results, with increased pH amplifying the remineralizing effect [26]. Xylitol, although pH neutral, prevents pH drops and preserves the activity of salivary enzymes. In addition to the active agents, excipients influence remineralization so that CaCO_3_-based abrasives act both mechanically and chemically, unlike silica, which is inert [27]. Optimal performance occurs when remineralizing agents are combined with a favorable composition profile, alkaline abrasives, antimicrobial ingredients, and the absence of anionic detergents, resulting in multifunctional pastes that support both enamel and overall oral health [30].

For enamel surface morphology, SEM analysis provided visual confirmation of the degree of enamel restoration. The healthy control sample showed uniform prisms without porosity [36], while the demineralized enamel had a honeycomb appearance with dissolved prismatic centers, as can be seen in the images obtained. The fluoride paste formed a protective film with amorphous aggregates that partially covered the pores [27], but at high magnifications, depressions were still visible. Nanohydroxyapatite partially filled the prism bodies, smoothing the surface, but the honeycomb outline remained [34]. NovaMin produced the greatest change: the surface became smooth, with the excavations completely filled with new apatite [25]. With xylitol, SEM showed minor surface changes and modest deposits, without complete disappearance of pores, although other studies reported smoothing comparable to fluoride [9]. The results support that fluoride, HA, and especially NovaMin technology restore the microstructure more effectively than xylitol.

The microhardness results expressed as Vickers Hardness Numbers (VHN) support the compositional and morphological trends observed by EDX and SEM. The significant increases in VHN recorded in the fluoride-containing groups (P1, P3, and P4) indicate a measurable reinforcement of the outer enamel layer, consistent with the higher Ca and P values detected in these samples. The NovaMin formulation (P3) showed the highest hardness recovery, reflecting the formation of a dense mineral layer with reduced surface porosity, while the arginine–fluoride group (P4) reached comparable VHN values, suggesting that the presence of arginine may enhance fluoride-driven mineral deposition. The nano-hydroxyapatite/xylitol formulation (P5) produced moderate but significant increases in VHN, in agreement with its progressive but incomplete pore filling observed microscopically. By contrast, the CPP-ACP formulation (P2) resulted in minimal microhardness changes. Overall, the microhardness findings confirm that the degree of mechanical recovery largely follows the extent of mineral deposition and structural consolidation achieved by each formulation.

The efficiency ranking shows NovaMin and fluoride paste at the top, followed by HA, all with marked increases in mineral content and reduction in roughness. NovaMin combines direct ion supply with chemical protection, easily outperforming fluoride in vitro. HA confirms its potential as a non-toxic alternative to fluoride [36]. Xylitol and enzymes, although with modest short-term remineralizing effects, act preventively by reducing biofilm acidogenicity and supporting natural processes. In vivo, their prolonged use can reduce the occurrence of lesions [16,37,38,39]. Combinations of agents (fluoride + HA, fluoride + xylitol, fluoride + enzymes) offer synergistic effects, minimizing the required dose of fluoride [30]. Our results show that toothpastes with F, HA, or NovaMin can effectively remineralize enamel, and in the long term, fluoride remains the gold standard in remineralization, although the alternatives studied remain viable options that require validation through extensive clinical studies.

The main limitation of the present study derives from its in vitro nature, which cannot fully reproduce the complexity of the oral environment, including behavioral factors, diet, pH variations, and interaction with bacterial biofilm. Furthermore, the 28-day testing period, although sufficient for observing remineralization trends, does not allow for the assessment of the long-term sustainability of the effect or the resistance of the enamel to repeated acid challenges. Individual variability in saliva composition and response to remineralizing agents could not be investigated in this experimental model. While SEM–EDX provides surface chemical information, Vickers microhardness testing offers a functional mechanical perspective; however, both methods are inherently limited to surface-related effects and do not reflect volumetric remineralization.

In the future, randomized clinical studies with extended follow-up periods are needed, including functional assessments such as abrasion resistance and correlating structural changes with relevant clinical parameters, to validate the transferability of the results obtained in vitro to current dental practice.

## 5. Conclusions

The therapeutic toothpastes demonstrated different remineralization abilities. Formulations containing fluoride, NovaMin, or arginine showed the most consistent improvements across EDX, SEM, and Vickers microhardness (VHN).

The NovaMin toothpaste (P3) achieved the highest Ca and P increases, a compact surface morphology, and the largest VHN gain. The arginine–fluoride paste (P4) produced comparable mineral recovery and hardness values.

The nano-hydroxyapatite/xylitol prototype (P5) yielded moderate mineral deposition and partial surface restoration, reflected in corresponding VHN increases. The conventional fluoride toothpaste (P1) produced significant but less pronounced effects.

The CPP-ACP formulation (P2) showed minimal changes in composition, morphology, and VHN.

Overall, formulations providing bioavailable calcium, phosphate, or fluoride ions demonstrated the greatest remineralization potential in this in vitro model.

These findings are limited to controlled in vitro conditions and should not be directly extrapolated to clinical performance without further in vivo and clinical validation

## Figures and Tables

**Table 1:** Composition of each toothpaste tested.

Group	Toothpaste	Composition	Manufacturer
**P1**	Sensodyne AntiCarie	Water, sorbitol, hydrated silica, glycerin, potassium nitrate, cocamidopropyl betaine, flavor, zinc citrate, xanthan gum, titanium dioxide, sodium fluoride, sodium hydroxide, sodium saccharin, sucralose, limonene. Contains sodium fluoride 0.315% *w*/*w* (1450 ppm fluoride).	GlaxoSmithKline (Brentford, UK)
**P2**	MI Paste/CPP–ACP (Recaldent™)	Water, Glycerol, D-Sorbitol, Casein Phosphopeptide–Amorphous Calcium Phosphate (CPP–ACP), CMC, Propylene Glycol, Titanium Dioxide, Xylitol, Phosphoric Acid, Flavor, Sodium Saccharin.	GC Corporation (Tokyo, Japan)
**P3**	Sensodyne Repair & Protect	Novamin Technology. Calcium Sodium Phosphosilicate 5%, Sodium Fluoride (1426 ppm), Glycerin, PEG 8, Hydrated Silica, Cocamidropropyl Betaine, Sodium Methyl Cocoyl Taurate, Aroma, Titanium Dioxide, Carbomer, Sodium Saccharin.	GlaxoSmithKline (Brentford, UK)
**P4**	Elmex Sensitive Professional	Arginine (8%). Calcium carbonate, aqua, sorbitol, bicarbonate, sodium lauryl sulfate, sodium monofluorophosphate (1450 ppm F), aroma, cellulose gum, sodium bicarbonate, tetrasodium pyrophosphate, titanium dioxide, benzyl alcohol, sodium saccharin, xanthan gum, limonene.	GABA International AG (Therwil, Switzerland)
**P5**	Experimental Hydroxyapatite and xylitol-based paste	Aqua, Mentha Piperita Flower/Leaf/Stem Water, Thymus Serpyllum Flower/Leaf/Stem Water, Laurus Nobilis Leaf Water, Glycerin, Xanthan Gum, Xylitol, Calcium Carbonate, Lithothamnion Calcareum Powder, Nano-hydroxyapatite, Zinc Citrate, Spirulina Platensis Powder, Decyl Glucoside, Sodium Benzoate, Potassium Sorbate, Laurus Nobilis Leaf Oil, Eugenia Caryophyllus Bud Oil, Melaleuca Alternifolia Leaf Oil.	Prototype (Faculty of Chemical Engineering and Environmental Protection “Cristofor Simionescu”, “Gheorghe Asachi” Technical University of Iaşi, Iasi, Romania)

**Table 2:** pH values of the tested toothpaste solutions.

Group	Toothpaste	pH Value
P1	Sensodyne AntiCarie	7
P2	MI Paste–CPP–ACP	7.04
P3	Sensodyne Repair & Protect	8.45
P4	Elmex Sensitive Professional	8.81
P5	Toothpaste with hydroxyapatite and xylitol	7.5

**Table 3:** Representative EDX elemental graph and SEM images of the morphological appearance of the enamel surfaces of a sample from each group at the end of days 1, 14, and 28 of testing.

Group	Day 1	Day 14	Day 28
P1 (Sensodyne Anticarie)	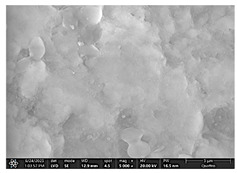	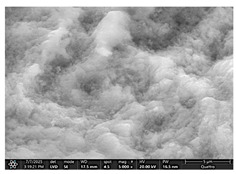	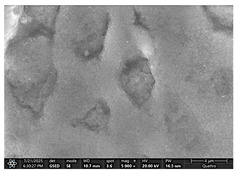
	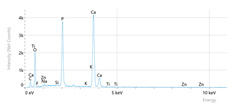	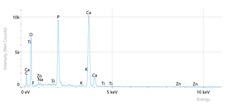	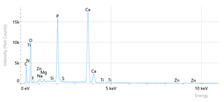
P2 (MI Paste/CPP-ACP)	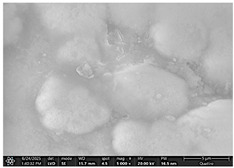	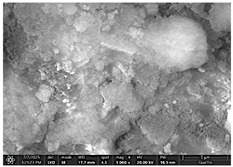	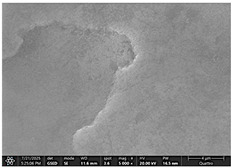
	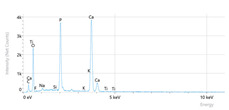	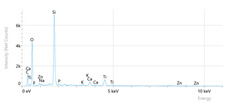	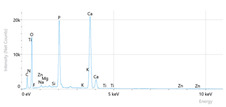
P3 (Sensodyne Repair & Protect)	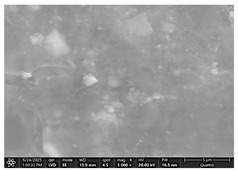	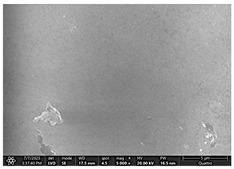	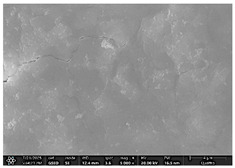
	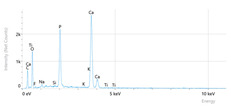	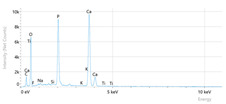	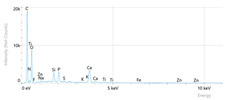
P4 (Elmex Sensitive Professional)	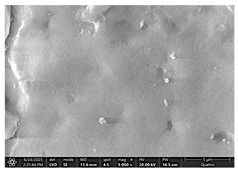	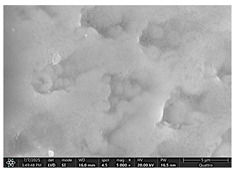	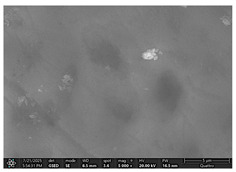
	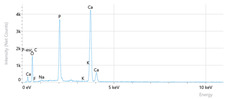	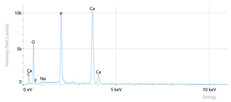	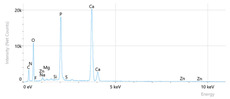
P5 (Hydroxyapatite Prototype, Xylitol)	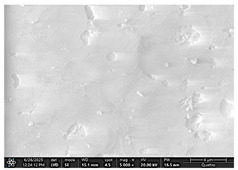	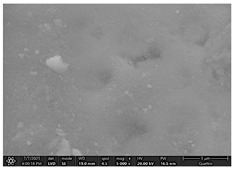	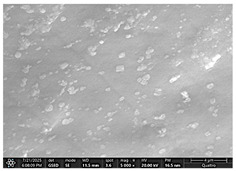
	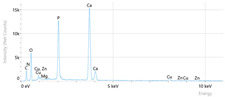	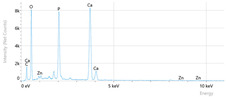	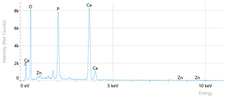
C1 (control)	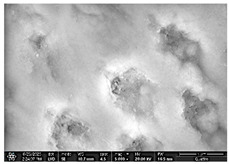	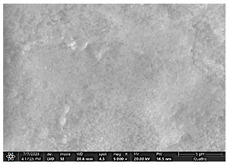	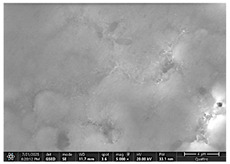
	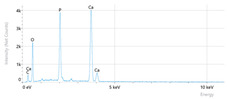	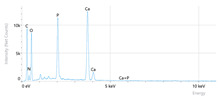	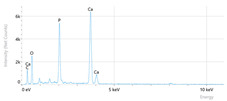

**Table 4:** Vickers microhardness values (VHN) at days 1, 14, and 28 (mean ± SD).

Group	Day 1 (HV)	Day 14 (HV)	Day 28 (HV)	Statistical Summary
C1	212 ± 18	216 ± 27	219 ± 14	No significant changes (*p* > 0.05)
P1 (Fluoride)	218 ± 20	292 ± 24	368 ± 17	Day 14 and 28 > Day 1 (*p* < 0.01); Day 28 > C1 (*p* < 0.001)
P2 (CPP-ACP)	215 ± 19	230 ± 21	252 ± 24	Not significantly different from C1 at any time (*p* > 0.05)
P3 (NovaMin)	225 ± 31	328 ± 26	405 ± 38	All timepoints significantly different (*p* < 0.001); highest at day 28 (*p* < 0.01)
P4 (Arginine + Fluoride)	221 ± 10	305 ± 35	387 ± 21	Increases at day 14 and 28 vs. day 1 (*p* < 0.01)
P5 (HA + Xylitol)	217 ± 29	265 ± 13	335 ± 16	Improvement of values (*p* < 0.05); Day 28 > C1 (*p* = 0.02)

## Data Availability

All the data presented in this study are available within the article.

## Abbreviations

The following abbreviations are used in this manuscript:

At.%	Atomic Percentage
Ca	Calcium
Ca/P ratio	Calcium-to-Phosphate Ratio
CO_2_	Carbon Dioxide
EDX/EDS	Energy-Dispersive X-ray Spectroscopy
F	Fluoride
HA	Hydroxyapatite/Nano-hydroxyapatite
Na	Sodium
NovaMin	Proprietary Calcium Sodium Phosphosilicate Technology
O	Oxygen
PEG	Polyethylene Glycol
SEM	Scanning Electron Microscopy
